# Sir Thomas Lauder Brunton, F.R.S. (1844-1916) about his visit to Hyderabad - Deccan: His role in the 2^nd^ Hyderabad Chloroform Commission (1889 A.D.)

**DOI:** 10.4103/0019-5049.71040

**Published:** 2010

**Authors:** Narayana Ala, A Ramachari, R Krishna Kumar

**Affiliations:** National Institute of Indian Medical Heritage and ESI Hospital, Hyderabad, India

## Abstract

Hundred years ago in Great Britain, Sir Thomas Lauder Brunton was acclaimed as an authority on the action of drugs on the heart. His famous work, “Pharmacology and Therapeutics” (1867), was read and appreciated all over Europe and America. He stated that nitrite of amyl has a beneficial effect on the heart. This led to the discovery of many other nitrite compounds that are now used for the treatment of angina pectoris. He also stated that mitral stenosis should be treated by surgery, i.e. by splitting the stenosed cusps. This statement paved the way for cardiac surgeons to devise the technique of closed mitral valvotomy. The results were encouraging and closed mitral valvotomy became the sheet anchor of treatment for a majority of the cases of mitral stenosis.

Lauder Brunton came to Hyderabad on 21^st^ October 1889 to participate in the deliberations of the 2^nd^ Hyderabad Chloroform Commission as an expert deputed by the editor of “The Lancet” to supervise the experiments regarding safety of chloroform as a general anaesthetic.[1] The Nizam (king) of Hyderabad, Mir Mehboob Ali Khan, sent £1,000/- to the editor of “The Lancet” to meet the travel expenses of Lauder Brunton.

A controversy was raging all over the world at that time regarding the safety of chloroform as a general anaesthetic. Hyderabad chloroform – Cap and technique of administering chloroform anaesthesia in Hyderabad became famous all over the world. He observed the administration of chloroform anaesthesia in Afzalgunj Hospital (Osmania General Hospital) in Hyderabad by the students of Hyderabad Medical School (Osmania Medical College). He appreciated the technique advocated by the surgeon, Major Edward Lawrice, who was Chief Surgeon and Principal of the Hyderabad Medical School. Letters written by Lauder Brunton eulogizing the method of administration of chloroform here were published in the leading Medical Journal, “The Lancet”, in 1890.

Experiments were conducted on dogs and monkeys to study the action of chloroform on the heart and respiration [Figure 1]. He recorded the blood pressure by catheterizing the carotid artery.[2] The graphs that he recorded on Ludwig’s and Ficks’s kymographs are still available for study and have been published[1] in the book, “Report on Hyderabad Chloroform Commission”, which was published in the year 1891.[1] Eminent members of the medical profession and nobles of Nizam’s court lavishly entertained him. The photographs [Figures 2 and 3] taken on these occasions are available in the National Institute of Indian Medical Heritage (formerly known as the Indian Institute of History of Medicine) in Osmania Medical College, Hyderabad.

**Figure 1:**
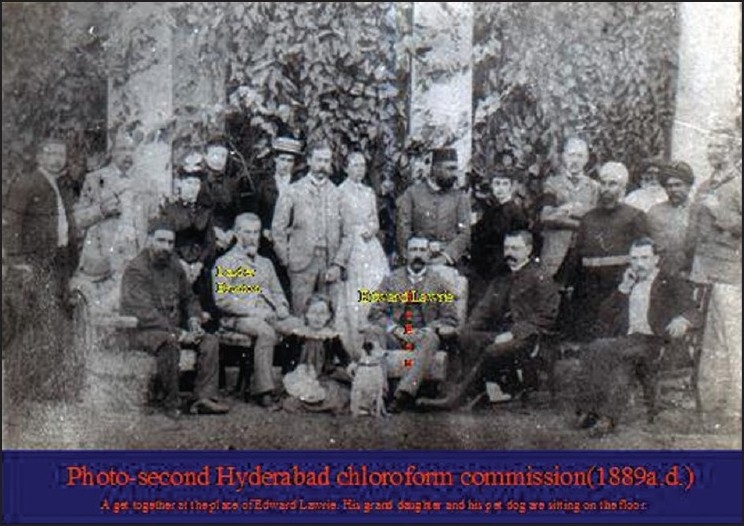
Hyderabad Chloroform Commission

**Figure 2:**
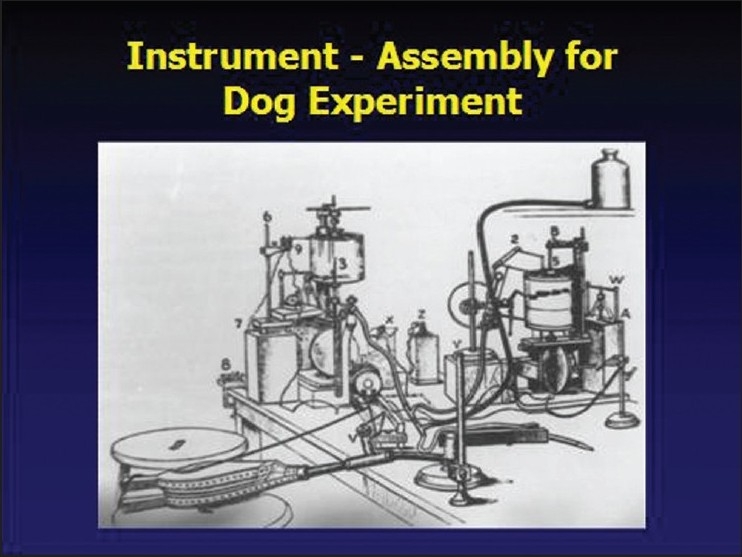
Assembly for the dog experiment

**Figure 3:**
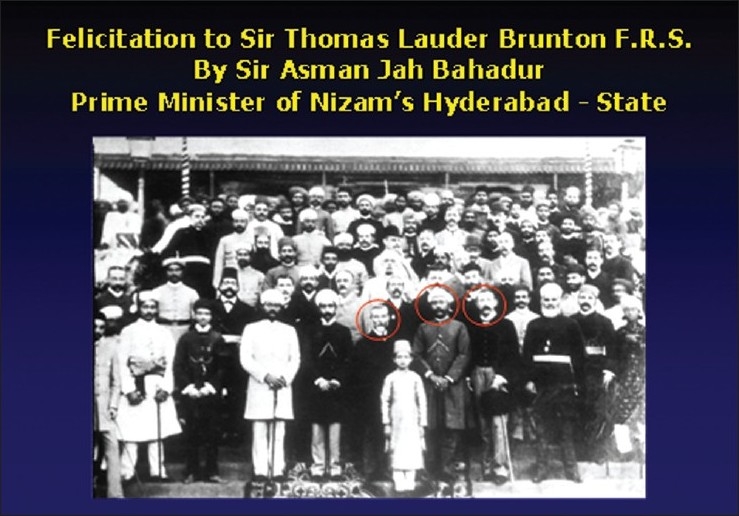
Felicitation to Sir Thomas Lauder Brunton

He left Hyderabad after a 57-day stay, on 18^th^ December 1889, but cherished the memories of the lavish hospitality of Hyderabad forever.
